# Troubleshooting of Endoscopic Ultrasound‐guided Rendezvous Using a Nasobiliary Drainage Tube

**DOI:** 10.1002/deo2.70237

**Published:** 2025-11-02

**Authors:** Tomohiro Yamazaki, Kenji Nakamura, Yuichiro Suzuki, Yuntae Kim, Shuhei Okuyama, Koichi Takagi, Katsuyuki Fukuda

**Affiliations:** ^1^ Department of Gastroenterology St. Luke's International Hospital Tokyo Japan; ^2^ Department of Gastroenterology Tokyo Dental College Ichikawa General Hospital Ichikawa Chiba Japan

**Keywords:** endoscopic ultrasound, EUS‐RV, nasobiliary drainage tube, rendezvous technique, troubleshooting

## Abstract

Endoscopic ultrasound‐guided rendezvous (EUS‐RV) is an alternative technique for patients in whom selective bile duct cannulation (SBDC) has failed during endoscopic retrograde cholangiopancreatography (ERCP). However, EUS‐RV has several challenging steps. Herein, we present a method for troubleshooting the EUS‐RV using a nasobiliary drainage tube (NBD) in a patient with a large periampullary diverticulum (PAD) and severe gastroptosis. An 80‐year‐old woman presented with nausea. Contrast‐enhanced computed tomography revealed a common bile duct (CBD) stone. Although ERCP was performed twice, the ampulla of Vater (AV) could not be identified due to the large PAD. Therefore, EUS‐RV was performed. The CBD was punctured from the descending part of the duodenum. Although a guidewire was advanced through the AV, the PAD hindered guidewire insertion to the anal side of the duodenum. During the switch to duodenoscopy, the guidewire was withdrawn due to gastroptosis. A subsequent attempt to puncture the CBD through the duodenal bulb resulted in guidewire entrapment. To manage the prolonged procedure, a 5‐French NBD was temporarily placed in the CBD. An NBD was subsequently inserted into the duodenum via the PAD using esophagogastroduodenoscopy under fluoroscopic guidance after 1 week. After switching to duodenoscopy, SBDC was successful along the NBD that was not withdrawn, and the stone was removed. NBD use in EUS‐RV may be effective in difficult cases of guidewire manipulation into the distal duodenum due to PAD and guidewire maintenance due to gastroptosis. Further, NBD is a readily available device, making its use convenient.

## 1 Introduction

The endoscopic ultrasound‐guided rendezvous (EUS‐RV) technique has become a therapeutic option when selective biliary duct cannulation (SBDC) fails during endoscopic retrograde cholangiopancreatography (ERCP), including cases with difficult identification of the ampulla of Vater (AV) due to a periampullary diverticulum (PAD) [1]. Although several ingenuities of the technique have been reported, guidewire manipulation and its maintenance in the biliary duct (BD) while switching to duodenoscopy are particularly challenging [2]. Furthermore, the lack of dedicated devices for EUS‐RV makes this procedure difficult. Herein, we report a method for troubleshooting the EUS‐RV using a nasobiliary drainage tube (NBD) for a difficult case of guidewire manipulation.

### 1.1 Case Report

**FIGURE 1:**
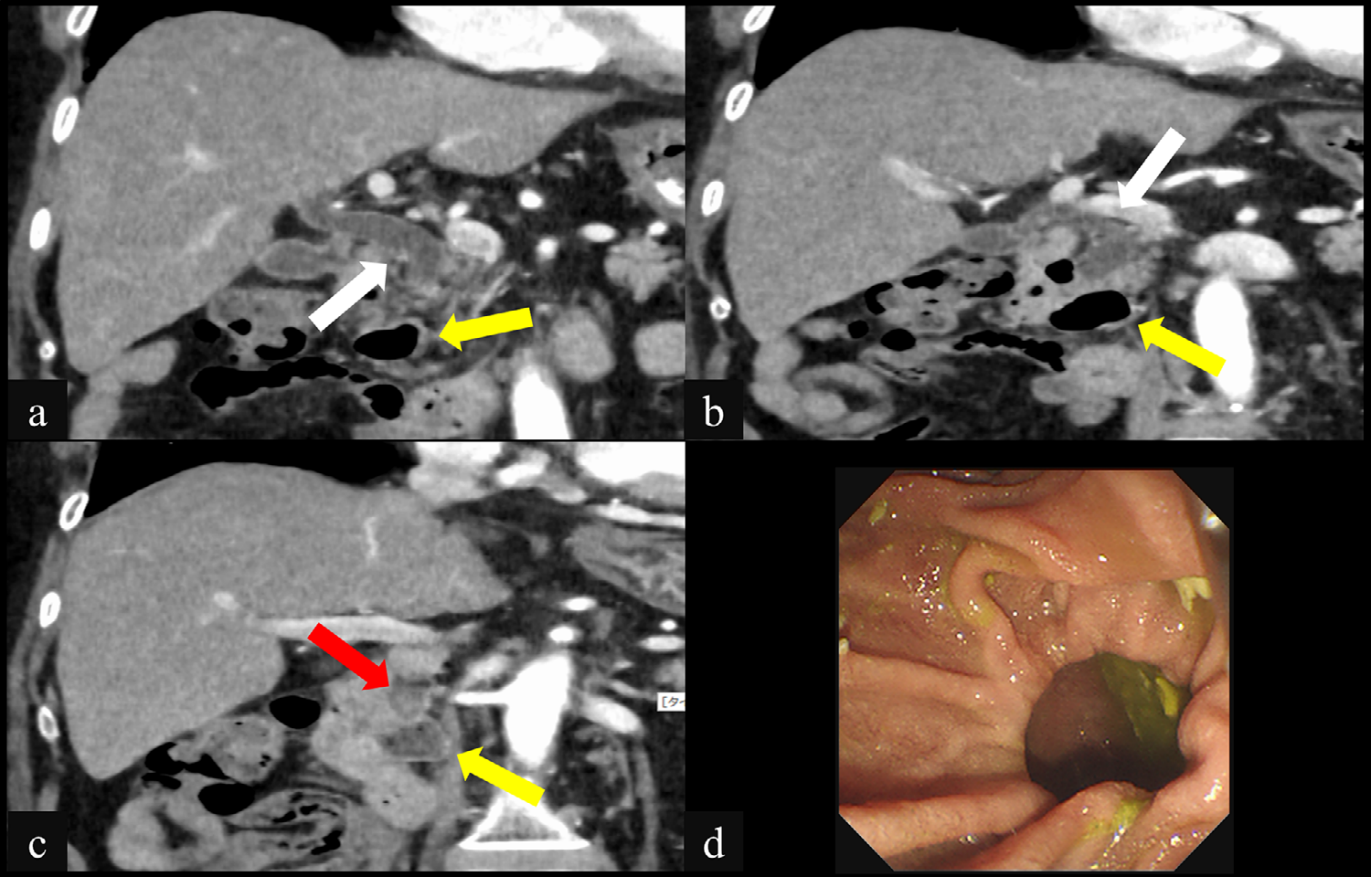
(a–c) Coronal images of contrast‐enhanced computed tomography at admission showing the choledocholithiasis (red arrows), periampullary diverticula (yellow arrows), and dilation of the extrahepatic bile duct (white arrows), and (d) Endoscopic views of the duodenoscopy showing periampullary diverticula with deep space and difficult confirmation of the orifice of the papilla.

**FIGURE 2:**
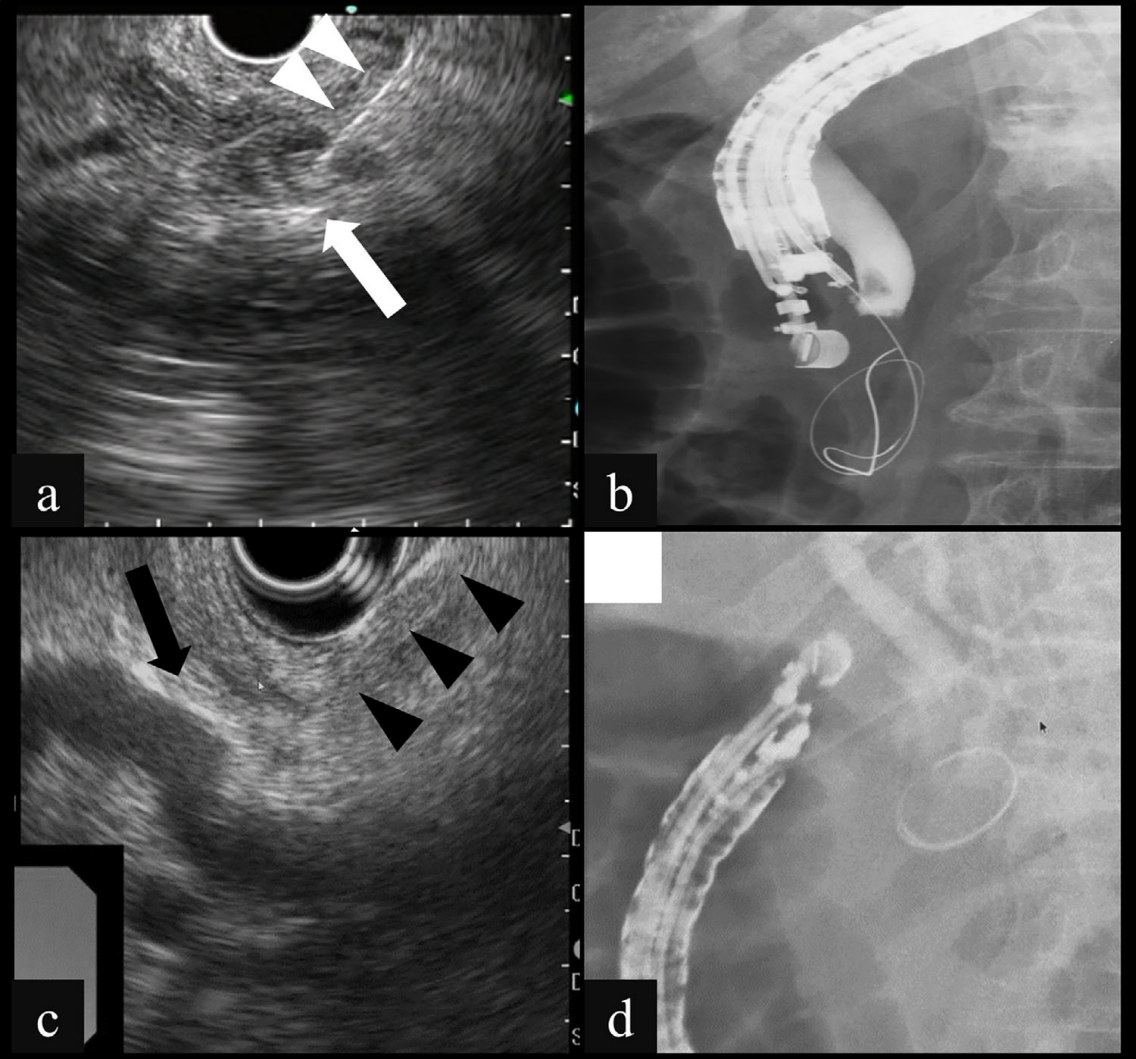
(a) Endoscopic ultrasound showing puncture of the lower bile duct (white arrow) using a 19‐gauge needle (white arrowheads) at the short scope position. (b) Fluoroscopy showing manipulation of the guidewire from the lower bile duct through the ampulla of Vater to the duodenum. (c) Endoscopic ultrasound showing puncture of the middle biliary duct (black arrow) using a 19‐gauge needle (black arrowheads) at the long scope position. (d) Fluoroscopy showing manipulation of the guidewire from the middle bile duct and guidewire entrapment within the periampullary diverticula.

**FIGURE 3:**
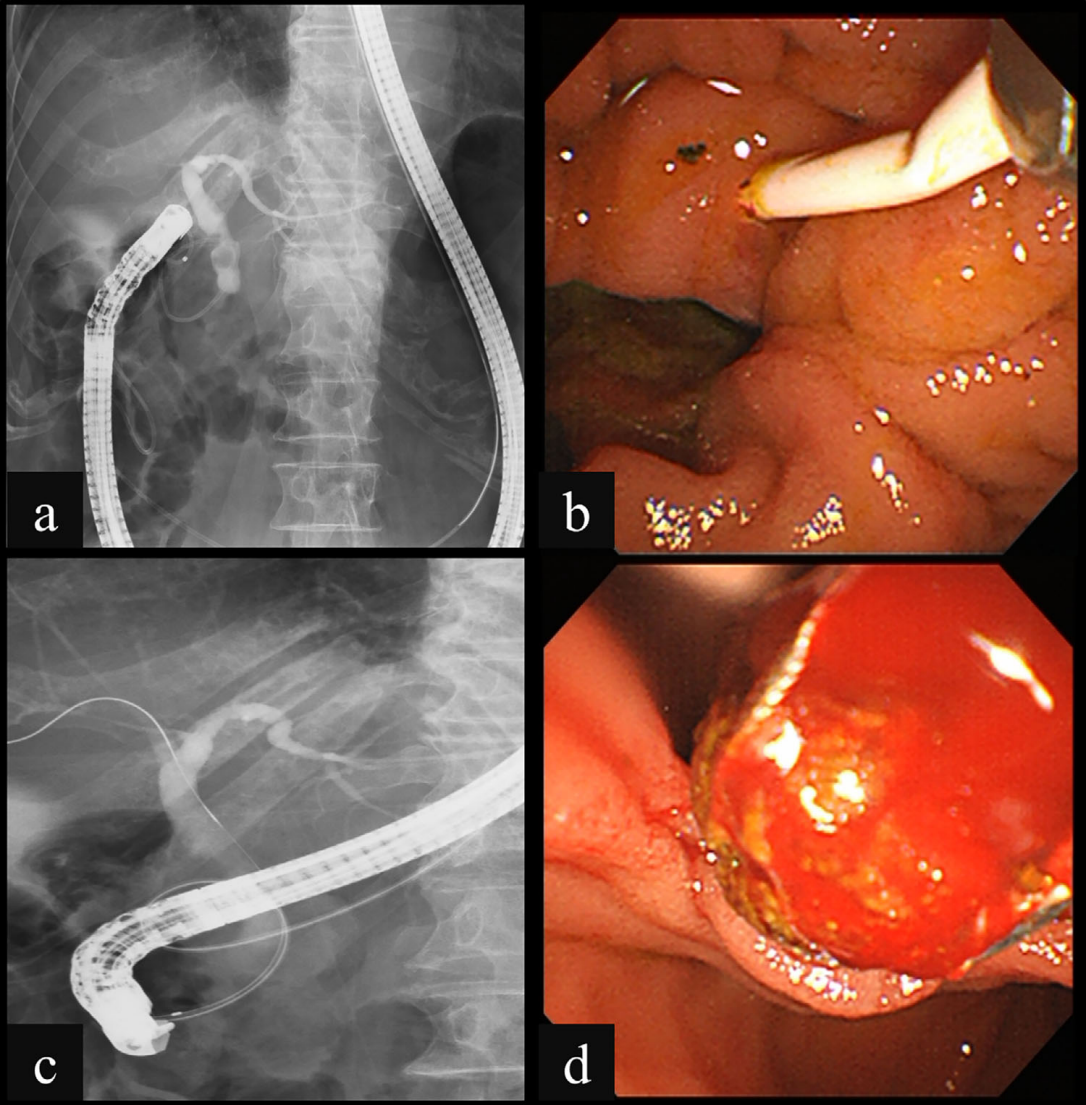
(a) Fluoroscopy showing the nasobiliary drainage tube (NBD) remaining in the duodenum despite severe gastric hyperextension during insertion of a duodenoscopy. (b) Duodenoscopy showing the ampulla of Vater, which was confirmed using the NBD as an indicator. (c) Fluoroscopy showing successful selective biliary duct cannulation guided by an NBD placed by the endoscopic ultrasound‐guided rendezvous technique. (d) Duodenoscopy showing successful removal of the biliary stone using a basket catheter.

An 80‐year‐old woman was referred to our hospital with nausea and elevated liver enzymes. The patient's vital signs on admission were blood pressure, 160/87 mmHg; pulse, 78/min; temperature, 35.9°C. Laboratory evaluation revealed elevated liver enzymes (total bilirubin, 2.8 mg/dL; direct bilirubin, 2.1 mg/dL; alkaline phosphatase, 372 IU/L; gamma‐glutamyl transpeptidase, 525 IU/L; aspartate aminotransferase, 101 IU/L; and alanine aminotransferase, 324 IU/L) and an elevated C‐reactive protein level of 5.44 mg/dL. Contrast‐enhanced computed tomography revealed a common bile duct (CBD) stone. The patient was diagnosed with acute cholangitis due to CBDs and subsequently hospitalized (Figure 1a–c). Although ERCP was performed twice, with esophagogastroduodenoscopy (EGD) during the second attempt, the AV could not be identified due to the large PAD (Figure 1d). After improvement of acute cholangitis with antibiotics, EUS‐RV was performed for stone removal. The lower BD was punctured from the descending part (D2) of the duodenum using a 19‐gauge needle under EUS guidance. A 0.025‐inch guidewire was advanced through the AV into the duodenum. However, the PAD hindered guidewire insertion into the anal side of the duodenum. During the switch to a duodenoscopy, the guidewire was withdrawn due to hyperextension of the stomach. A subsequent attempt to puncture the BD through the duodenal bulb (D1) resulted in guidewire entrapment within the PAD (Figure 2). To manage the prolonged procedure, our therapeutic strategy changed to a two‐stage procedure, and a 5‐French (Fr) NBD was temporarily placed in the BD after dilation with a 7‐Fr dilator as in EUS‐guided choledochoduodenostomy (EUS‐CDS). At 1 week after EUS‐RV, the absence of contrast leakage outside the BD was confirmed by contrast examination through NBD performed the day before. The EGD was inserted through the NBD, which was switched from the nose to the oral route, to the puncture site under fluoroscopic guidance. Then, a guidewire was inserted into the NBD and duodenum, and the NBD was directed through the PAD to the duodenum following the guidewire. After switching to duodenoscopy, the NBD remained in the BD despite hyperextension of the stomach. SBDC was successful along the NBD, and the stone was removed following endoscopic sphincterotomy (EST) (Figure 3). The patient was discharged 4 days post‐procedure without complications.

The schema and equipment for our procedure are shown in Figure S1 and Table S1.

## 2 Discussion

ERCP and related procedures are the first choice of treatment for symptomatic CBDs. CBDs cannot be removed unless SBDC is achieved during ERCP. The factors related to difficulties in performing SBDC include physician factors, such as experience, and patient factors, such as anatomical variants, including PAD. Recent studies have reported that PAD does not affect the success rate of SBDC. However, they also reported that the time to achieve SBDC is longer in patients with PAD, particularly type‐I PAD [3]. Several advanced techniques have been reported for patients with PAD in whom SBDC is difficult, including double guidewire cannulation, pancreatic stenting with pre‐cut sphincterotomy, and cap‐ or traction‐assisted cannulation. When these are unsuccessful, PTB‐RV or EUS‐RV may be considered as rescue options. PTB‐RV can be performed via the intrahepatic bile duct and is easier if a drainage tube is present [4].

EUS‐RV is another effective approach, despite limitations such as the need for advanced skills and the lack of dedicated devices. It can be classified into four types by puncture and rendezvous route [5], and the advantages and disadvantages of each are shown in Table 1. Device‐related improvements, including microcatheters and recessed contrast catheters to secure guidewire position, have also been proposed [6, 7].

**TABLE 1:** Comparison of endoscopic ultrasound‐guided rendezvous techniques using four different puncture sites.

	EHBD			IHBD
Puncture site	D1	D2	Stomach	Stomach
Schema	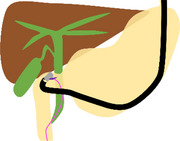	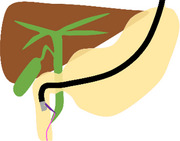	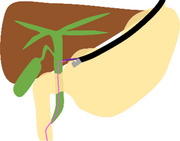	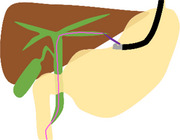
Scope position	Long	Short	Short	Short
Scope stability	Stable	Instable	Stable	Stable
Need to IHBD dilation	No	No	No	Yes (basically)
Handling the puncture needle	Sometimes difficult	Sometimes difficult	Easy	Easy
Needle direction	Hepatic hilar	AV	AV	AV
Distance to the AV	Short	Very short	Short	Long
Guidewire manipulation to the AV	Sometimes difficult	Easy	Easy	Sometimes difficult
Guidewire stability during scope exchange	Poor	Poor	Good	Good
Intervening parenchymal organs	None	Pancreas	None	Liver
Risk of bile leak	High	Low to moderate	High	Low

Abrreviations:: AV, ampulla of Vater, D1, duodenal bulb, D2, descending part of the duodenum, EHBD, extrahepatic bile duct, IHBD, intrahepatic bile duct.

In our case, no dilatation of the IHBD (<2 mm) was observed; therefore, PTB‐RV or endoscopic ultrasound‐guided hepaticogastrostomy (EUS‐HGS) was not the priority route, and we first approached from D2. However, insertion of the guidewire into the anal side of the duodenum using a D2 approach was difficult because of entrapment inside the PAD. Although the use of a 3‐Fr microcatheter from the D2 approach has been reported as effective in such situations, this device was not available in our institution [8]. Instead, we adopted a troubleshooting strategy using a nasobiliary drainage (NBD) catheter, which is more commonly available. The duodenoscopy was replaced with the guidewire inside the PAD; however, the guidewire was dislodged. Therefore, we switched to a D1 approach. However, we were unable to properly manipulate the guidewire, and the procedure was prolonged. Therefore, we managed this case with NBD to prevent postoperative complications such as bile leakage and to facilitate a second procedure by placing NBD. Although the guidewire manipulation was difficult and the guidewire was trapped inside the PAD, the NBD could easily pass through the PAD, which helps support SBDC. When placing the NBD, the D2 approach would require placement via the pancreatic parenchyma, and the D1 approach seemed to be a better site for NBD placement. If the NBD is inserted through the pancreatic parenchyma, pancreatitis or pancreatic fistula may occur because of damage to the pancreatic parenchyma or the branch duct. Therefore, the D1 or transgastric route may be better for this procedure. However, neither route passes through parenchymal organs, and unless a fistula is formed, a high risk of bile leakage exists, which should be managed cautiously.

We repeated the procedure 1 week after the fistula was considered to be mature. After guiding the NBD to the duodenum using the EGD under fluoroscopic guidance, SBDC was successfully performed along the NBD, and the CBD was removed following EST.

The procedure was performed 1 week after NBD placement, although clear evidence on adequate fistula maturation time is lacking. For interventional EUS (I‐EUS), no studies have addressed EUS‐CDS; however, in one report using EUS‐HGS with antegrade stone removal, a stent was placed to prevent bile leakage and removed after 1–2 weeks [9]. One reports exist on stone removal at ≥1 month post‐procedure [10], and further studies are warranted. To minimize the risk of bile leakage, we placed a thin 5‐Fr drainage tube. Contrast examination via NBD the day before the second procedure showed no extrabiliary leakage, and no leakage was observed during the stone removal or after transpapillary biliary stenting. The 5‐Fr NBD's flexibility allowed it to remain in position during endoscope exchange, even with gastric deformity. However, since it was placed after dilation with a 7‐Fr dilator, potential mismatch at the puncture site should be considered. In general, the tip of the NBD catheter should be placed on the hepatic side of the CBD when biliary drainage is the primary objective. However, in our case, to prevent bile peritonitis and facilitate easier guidance of the NBD catheter into the duodenum during the subsequent procedure, we intentionally placed the tip on the downstream side of the CBD in the BD. Despite several considerations, this approach may serve as a useful troubleshooting strategy in EUS‐RV. NBD use during EUS‐RV may be effective in difficult cases of guidewire manipulation into the distal duodenum due to PAD and guidewire maintenance due to gastroptosis. The NBD is not a specialized but rather a readily available device, making it a convenient option.

## Author Contributions

**Tomohiro Yamazaki** designed the study, collected and analyzed the data, and drafted the manuscript. **Kenji Nakamura** contributed to data interpretation and manuscript revision. **Yuichiro Suzuki**, **Yuntae Kim**, **Shuhei Okuyama**, and **Koichi Takagi** provided critical revision of the manuscript for important intellectual content. **Katsuyuki Fukuda** supervised and provided critical revision of the manuscript for important intellectual content. All authors read and approved the final manuscript.

## Conflicts of Interest

The authors declare no conflicts of interest.

## Funding

The authors have nothing to report.

## Ethics Statement

The authors report the details of the patient's case in accordance with the ethical standards of the Helsinki Declaration of 1975, as revised in 2008(5).

## Consent

Informed consent was obtained from the patient for this case report and accompanying images.

## Clinical Trial Registration

Not applicable.

## Supporting information




**FIGURE S1** Schema of the troubleshooting of endoscopic ultrasound‐guided rendezvous using a nasobiliary drainage tube (NBD). (a) The lower bile duct was punctured from the descending part using a 19‐gauge needle under endoscopic ultrasound (EUS) guidance, and a guidewire was advanced through the papilla into the duodenum via the bile duct (BD). (b) The EUS was removed while the guidewire was placed in the duodenum. (c) During switching to and insertion of the duodenoscope, the guidewire was withdrawn from the BD due to severe gastric hyperextension caused by gastroptosis. (d) A subsequent attempt to puncture the middle BD through the duodenal bulbus using EUS resulted in guidewire entrapment within the periampullary diverticulum. (e) The NBD was placed in the BD from the puncture site. (f) Under fluoroscopic guidance, an esophagogastroduodenoscopy was inserted through the NBD, which was switched from the nose to the oral route, and the NBD was placed through the running guidewire into the duodenum via the papilla. (g) Exchanging the guidewire for an NBD made the procedure more manageable, allowing successful advancement of the duodenoscope beyond the periampullary diverticula and into the duodenum. (h) Despite the severe gastric hyperextension, the NBD was retained in the BD, and selective biliary duct cannulation was successfully performed along the NBD.

**TABLE S1** Equipment in our procedures.
