# Supplementary figures and images for: Troubleshooting of Endoscopic Ultrasound‐guided Rendezvous Using a Nasobiliary Drainage Tube
Source: DEN Open. 2025 Nov 2;6(1):e70237. doi: 10.1002/deo2.70237 (PMC12580290; doi:10.1002/deo2.70237)

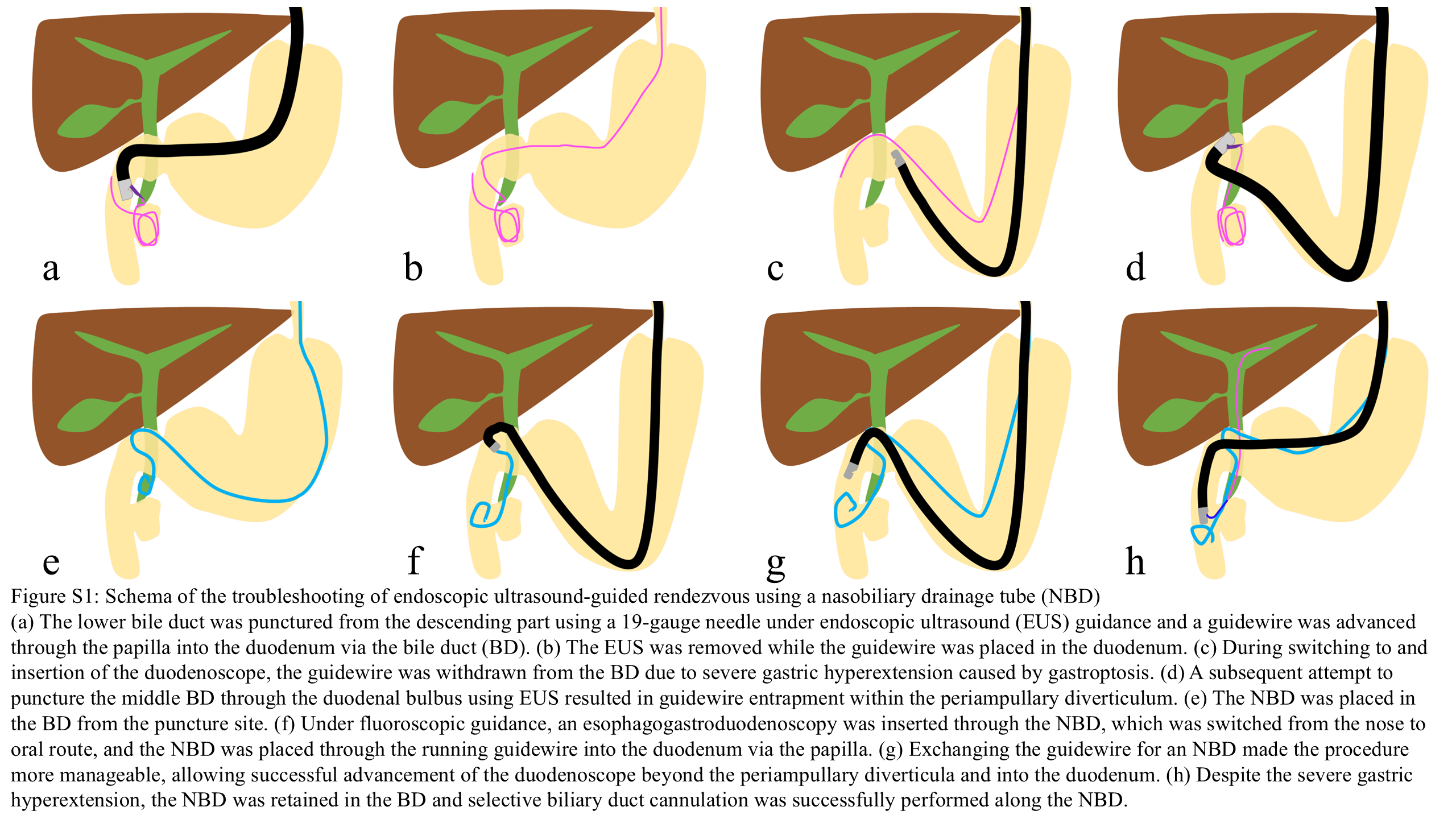

Supplement: Supplementary file 1 — FIGURE S1 Schema of the troubleshooting of endoscopic ultrasound‐guided rendezvous using a nasobiliary drainage tube (NBD). (a) The lower bile duct was punctured from the descending part using a 19‐gauge needle under endoscopic ultrasound (EUS) guidance, and a guidewire was advanced through the papilla into the duodenum via the bile duct (BD). (b) The EUS was removed while the guidewire was placed in the duodenum. (c) During switching to and insertion of the duodenoscope, the guidewire was withdrawn from the BD due to severe gastric hyperextension caused by gastroptosis. (d) A subsequent attempt to puncture the middle BD through the duodenal bulbus using EUS resulted in guidewire entrapment within the periampullary diverticulum. (e) The NBD was placed in the BD from the puncture site. (f) Under fluoroscopic guidance, an esophagogastroduodenoscopy was inserted through the NBD, which was switched from the nose to the oral route, and the NBD was placed through the running guidewire into the duodenum via the papilla. (g) Exchanging the guidewire for an NBD made the procedure more manageable, allowing successful advancement of the duodenoscope beyond the periampullary diverticula and into the duodenum. (h) Despite the severe gastric hyperextension, the NBD was retained in the BD, and selective biliary duct cannulation was successfully performed along the NBD. [file DEO2-6-e70237-s002.TIF]
