# Supplementary material for: Troubleshooting of Endoscopic Ultrasound‐guided Rendezvous Using a Nasobiliary Drainage Tube
Source: DEN Open. 2025 Nov 2;6(1):e70237. doi: 10.1002/deo2.70237 (PMC12580290; doi:10.1002/deo2.70237)
Supplement: Supplementary file 2 — TABLE S1 Equipment in our procedures. [file DEO2-6-e70237-s001.docx]

Equipment

|  | **Generic Name** | **Trade Name** | **Manufacturer** | **City / State** | **Country** |
| --- | --- | --- | --- | --- | --- |
| Endoscope | Esophagogastro-duodenoscopy | GIF-H290T | Olympus | Tokyo | Japan |
|  | Echoendoscope | GF-UCT260 | Olympus | Tokyo | Japan |
|  | Duodenoscopy | TJF-260V | Olympus | Tokyo | Japan |
| Device | Puncture needle | EZ shot 3 plus | Olympus | Tokyo | Japan |
|  | Guidewire | VisiGlide 2 | Olympus | Tokyo | Japan |
|  | Naso-biliary drainage tube | Silky Pass | Boston Scientific | Natick, MA | USA |
|  | Biliary drainage tube | Flexima | Boston Scientific | Natick, MA | USA |
|  | ERCP catheter | MTW | MTW | Hyogo | Japan |
|  | Stone extraction basket | Double Lumen Extraction Basket | Cook Medical | Winston-Salem, NC | USA |
